# Determination of Grain-Boundary Structure and Electrostatic Characteristics in a SrTiO_3_ Bicrystal by Four-Dimensional Electron Microscopy

**DOI:** 10.1021/acs.nanolett.1c02960

**Published:** 2021-10-21

**Authors:** Chao Yang, Yi Wang, Wilfried Sigle, Peter A. van Aken

**Affiliations:** † Max Planck Institute for Solid State Research, Stuttgart 70569, Germany; ‡ Center for Microscopy and Analysis, Nanjing University of Aeronautics and Astronautics, Nanjing 210016, P.R. China

**Keywords:** 4D-STEM, electrostatic characteristics, grain boundary, oxygen vacancy

## Abstract

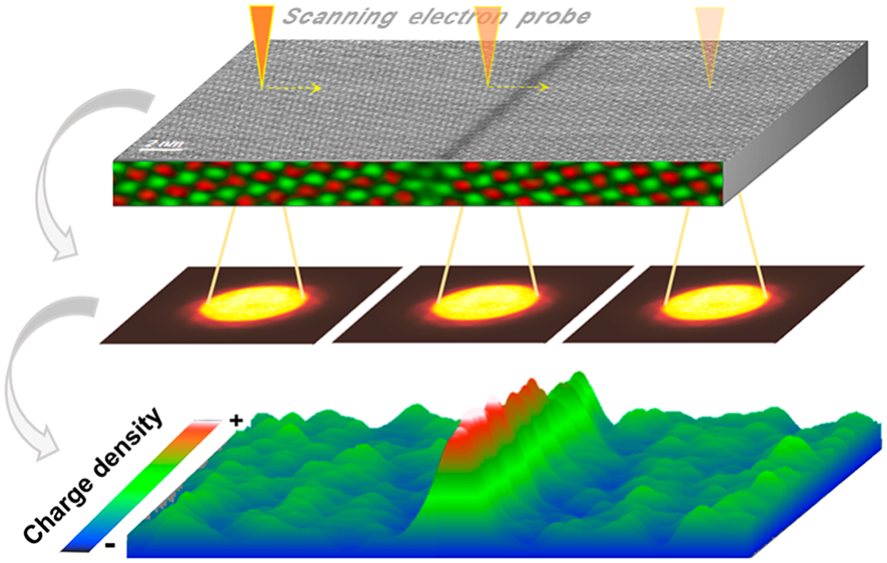

The grain boundary (GB) plays a critical role in a material’s properties and device performance. Therefore, the characterization of a GB’s atomic structure and electrostatic characteristics is a matter of great importance for materials science. Here, we report on the atomic structure and electrostatic analysis of a GB in a SrTiO_3_ bicrystal by four-dimensional scanning transmission electron microscopy (4D-STEM). We demonstrate that the Σ5 GB is Ti-rich and poor in Sr. We investigate possible effects on the variation in the atomic electrostatic field, including oxygen vacancies, Ti-valence change, and accumulation of cations. A negative charge resulting from a space-charge zone in SrTiO_3_ compensates a positive charge accumulated at the GB, which is in agreement with the double-Schottky-barrier model. It demonstrates the feasibility of characterizing the electrostatic properties at the nanometer scale by 4D-STEM, which provides comprehensive insights to understanding the GB structure and its concomitant effects on the electrostatic properties.

Direct imaging of electrostatic characteristics and determination of the magnitude and distribution of space charges at grain boundaries (GBs) has always been challenging and has attracted attention for decades. Inline and offline holographic techniques use phase-contrast imaging that is sensitive to electric potentials in solids. The phase is reconstructed from the scattered exit electron wave through the object, which enables imaging of the electrostatic potential with nanometer spatial resolution.^1−5^ More recently, differential phase-contrast imaging (DPC) has been used to reconstruct the electrostatic potential at atomic resolution through the difference signal of opposed detector segments. This difference signal is proportional to the beam deflection along the direction connecting the two segments.^6−9^ Shibata et al. described how the application of a segmented detector in DPC-STEM imaging enables a real-time reconstruction of the HAADF image, the electric-field-vector map, and the charge-density map and hence allows the simultaneous acquisition of the local atomic structure and electrostatic information from the same area.^7^ Further progress was achieved by the use of pixelated detectors instead of segmented detectors. Such pixelated detectors allow detection of a full diffraction pattern for every probe position on the specimen, also called 4D-STEM. With this, electrostatic field measurements have become possible on the atomic scale and a simplified quantum mechanical approach was proposed to measure electric fields.^10^ Gao et al. reported on the quantitative characterization of the electric field and charge density of an ultrathin SrTiO_3_/BiFeO_3_ heterojunction at subangstrom resolution by 4D-STEM.^11^ Additionally, 4D-STEM enables imaging the electric field at a large scale, for example, the polarization-induced internal electric field in 110 nm thick wurtzite AlN/GaN nanowires.^12^ Basically, most electrostatic-property studies focus on heterojunctions, interfaces, or point defects. The simultaneous characterization of the atomic structure and electrostatic characteristics in more complicated defects, for example, GBs in electroceramic materials, is critical and essential to explore the mechanisms of the electrical and mechanical behavior.

GBs play a dominant role in the electrical properties of many electroceramics. This is often related to nonstoichiometry or segregation, which can promote the formation of space charges near GBs. These effects can control electrical conductivity across and along GBs. During the past decades, the double-Schottky-barrier model has been established for electrically blocking grain boundaries in ionic solids, which explains the electrical behavior of GBs by the presence of a GB-core charge, whose electric field is compensated by a space charge on both sides of the GB.^1,13−17^ For example, positively charged defects, e.g., oxygen vacancies or excess cations at an acceptor-doped SrTiO_3_ GB, form a potential barrier. A negatively charged space-charge zone in the adjacent bulk regions compensates these positively charged defects.^18^ Generally, these descriptions via charged defects and space-charge layers are indirectly deduced from experimental data of bulk properties, e.g., from impedance spectroscopy or *I*–*V* properties.^13,19−22^

Here, we selected a SrTiO_3_ bicrystal with a Σ5 (310) GB with a tilt angle of the neighboring grains of 36.9° around the common [100] axis. This GB has been extensively characterized in the past by different techniques, however with partially contradicting results. This may be due to either different impurity levels of the used crystals or inadequate analysis of the data. It is generally accepted that the GB is not symmetric but shows a translation of the adjoining grains of less than 0.1 nm parallel to the GB plane, i.e., the ⟨310⟩ direction.^15,23^ However, in Kim et al.^24^ and Shao et al.,^19^ a symmetrical GB was assumed. Although early studies by energy-dispersive X-ray analysis^25,26^ seemed to show a stoichiometric GB, most later studies found the GB to contain defects and charge.^14,24,27,28^ Most importantly, it was found that the ratio of Ti-to-O is increased at the GB, and this is usually assigned to the need to remove oxygen atoms from the GB core because of their close proximity. Thus, the Ti valence is generally found to decrease at the GB core. It is this deficiency of oxygen ions in the GB core that leads to the translational asymmetry mentioned above. Additionally, the intermixing of cations at the GB is important to understand the microstructure of the GB.

In this work, we use the latest advances in TEM analysis, in particular the 4D-STEM technique in combination with advanced aberration-corrected STEM and EELS, to elucidate microstructure, chemical composition, and electrostatic characteristics of a Σ5 (310) GB in a SrTiO_3_ bicrystal at the nanometer and atomic scale. We discuss how the charged defects, the change of the Ti valence state, and the structural distortion affect the measured averaged momentum transfer and electrostatic field at the GB. This case study demonstrates the power of 4D-STEM for understanding the GB properties.

## Results

**Figure 1:**
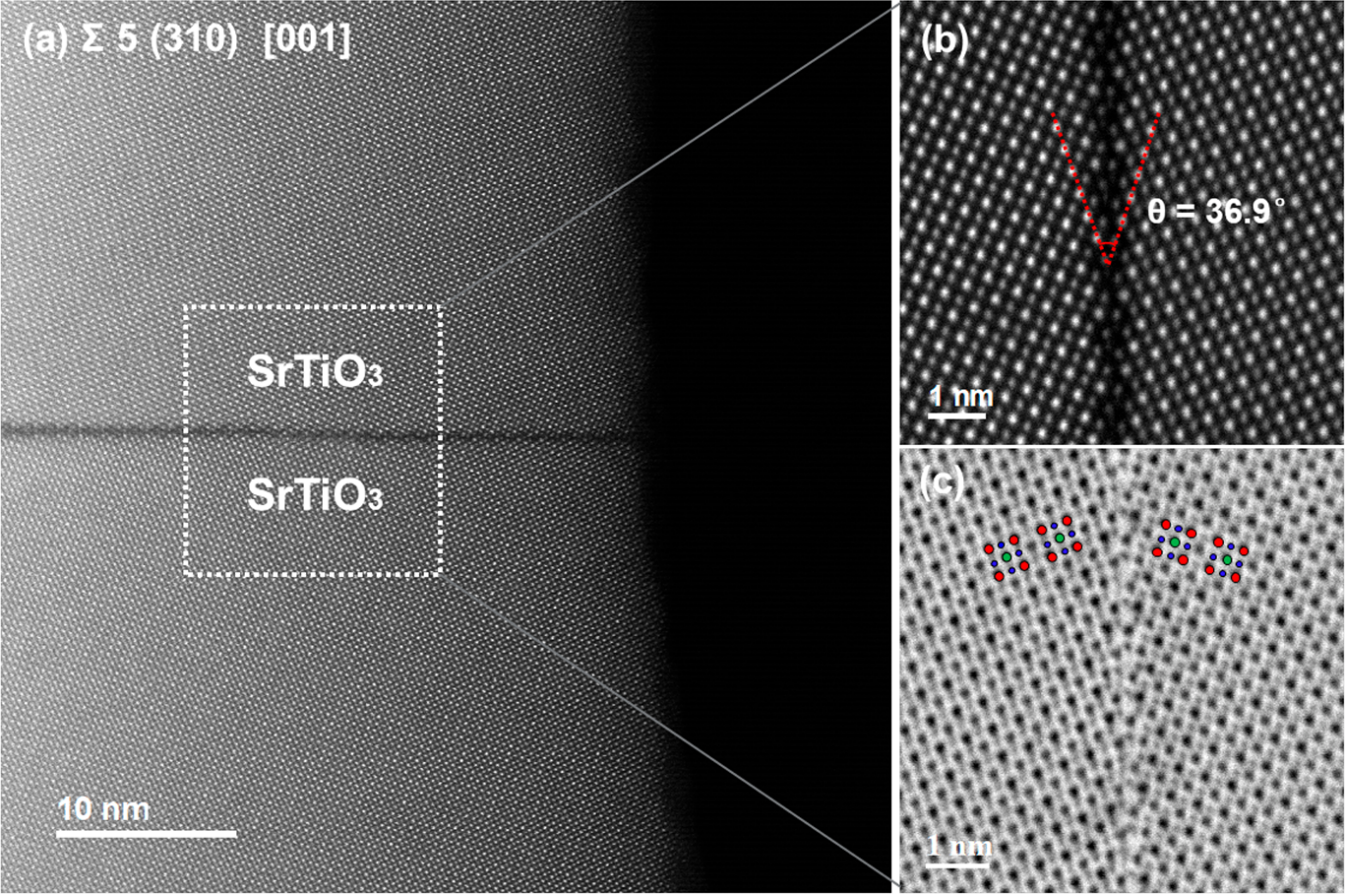
Microstructural characterization of the SrTiO_3_ bicrystal. (a) Overview HAADF-STEM image of the Σ5 (310) [001] STO bicrystal. Atomically resolved (b) HAADF-STEM image and (c) ABF-STEM image, where oxygen columns are clearly visible and the image is overlaid with projections of the STO unit cell (Sr, red; Ti, green; O, blue).

**Figure 2:**
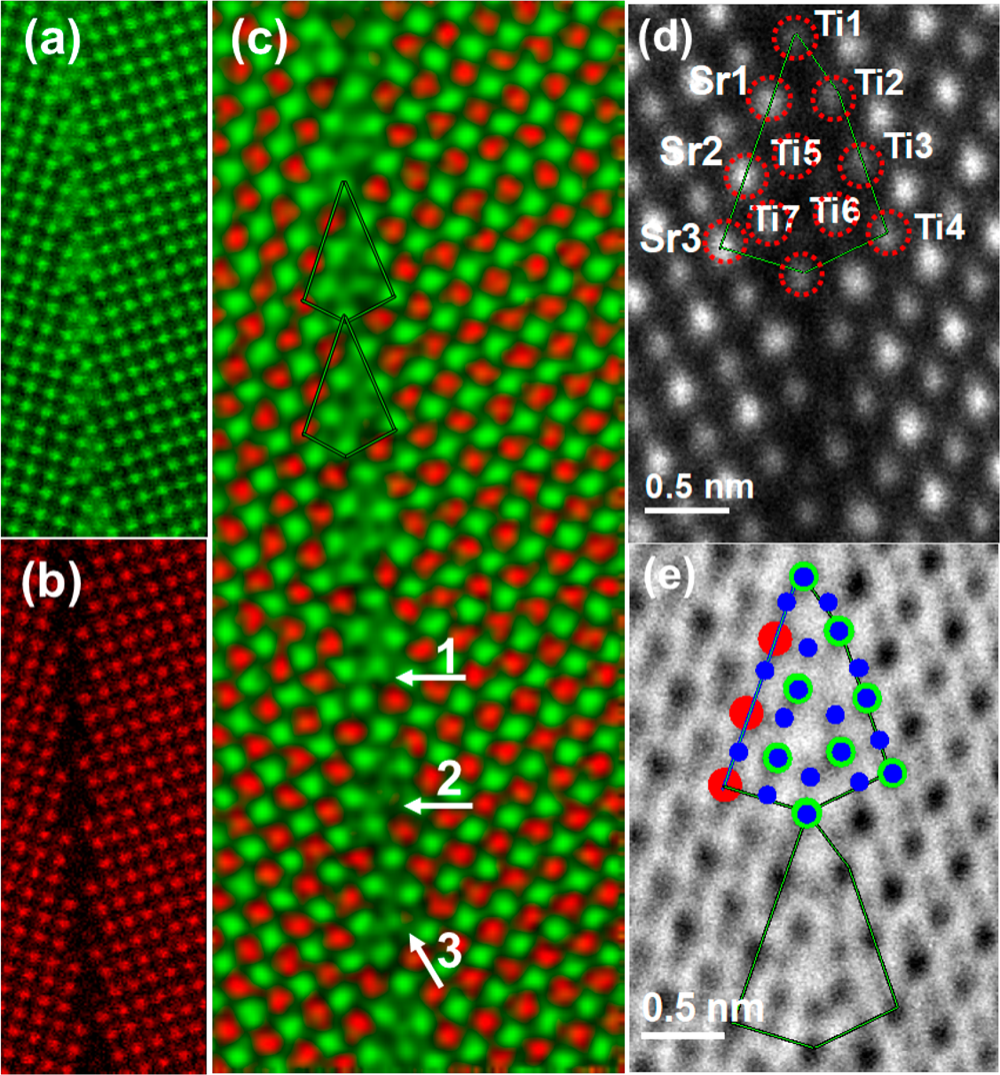
Elemental distribution at the Σ5 GB. EELS maps of (a) Ti and (b) Sr. (c) Color-coded mapping of Ti (green) and Sr (red). The white arrows indicate the atomic columns with intermixing of Sr and Ti. (d) ADF image at the GB. The cations of the GB core are marked with red dashed circles. (e) Experimental ABF image of the GB. The oxygen columns are marked with blue spots, Sr columns in red, and Ti columns in green.

Figure 1 shows atomically resolved STEM images of the Σ5 (310) [001] STO bicrystal, where Σ5 means that, if we imagine both crystals overlapping, every fifth atomic position would be common to the two lattices. Because of the translational asymmetry shown below, this geometrical reasoning is not strictly true anymore. The Σ5 GB has a (310) GB plane and a [001] rotation axis.^29^Figure 1a, b shows a uniform and high-quality GB structure. We can easily identify the Sr and mixed Ti–O columns in the bulk region from the HAADF-STEM image according to the relationship between the atomic number *Z* and the intensity of each atomic column.^30^ The tilt angle of the GB amounts to the expected value of 36.9°. We find a translation of the two lattices of 0.05 nm parallel to the GB plane, which is consistent with the reported values of 0.06 nm from HRTEM,^23^ 0.05 nm from DFT, and 0.07 nm from HAADF images.^15^ Because of the decreased atom contrast at the GB, we cannot distinguish the different elements from the HAADF image. The image contrast at the GB is weak, which is related to structural distortions, the sample thickness, and/or the presence of vacancies.^15^ From the corresponding ABF image in Figure 1c, the oxygen columns are clearly visible. This is the first attempt to image oxygen ions at a Σ5 GB in STO. In principle, not only cations but also anions are mirror symmetric with respect to the GB plane, if we ignore the small rigid-body translation.^15^ However, we detect the incomplete [TiO_6_] octahedra in Figure 2e rather than a rigid-body translation.

To determine the positions of the elements at the GB, we measured electron energy-loss spectra (EELS) and extracted atomic-resolution elemental maps to identify the different cations at the GB. Figure 2a–c depicts the distribution of Ti and Sr. We find that the GB is Ti-rich and Ti columns marked by white arrows contain minor amounts of Sr. In terms of the atomic information obtained from HAADF, ABF, and EELS mapping, we propose a possible structure of the GB core in Figure 2e. The red circles denote Sr, blue circles represent O, and green circles indicate Ti. Compared with a perfect Σ5 GB core structure, the Sr sites are almost occupied by Ti atoms in the periodic GB core structure verified by EELS maps. The proposed GB core structure is stable under the electron probe, which is unlike the reported dislocation core structure.^31^ In addition, we compared the relative signal intensities of Ti and O by tilting the grain boundary away from zone axis and found that the Ti/O ratio shows an increase in the GB compared to that in the bulk, demonstrating excess Ti ions and oxygen vacancies in the GB.

**Figure 3:**
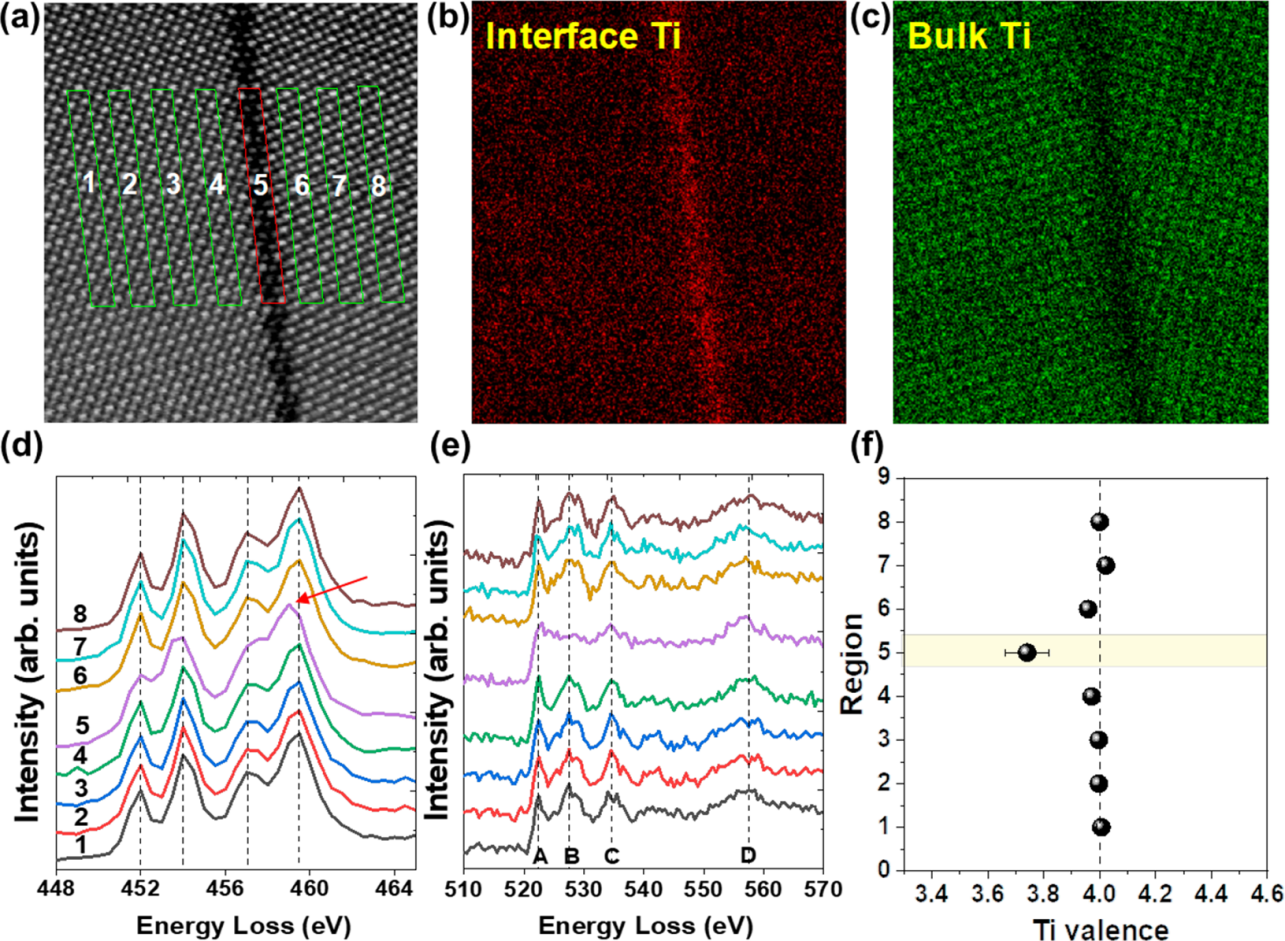
EELS measurements of the Ti-*L*_2,3_ and O-*K* edges at the GB. (a) Assignment of areas in the ADF image for EELS data analyses. Mapping of (b) interfacial Ti and (c) Ti in the bulk. (d) Averaged Ti-*L*_2,3_ white lines extracted from the regions marked in panel a. (e) Averaged O-*K* edges extracted from the regions marked in panel a. The letters A, B, C, and D designate the corresponding spectral features in the O-*K* spectra. The Ti-*L*_2,3_ and O-*K* spectra extracted at position 5 are from the interface. (f) Valence states of Ti calculated by the multiple-least-squares fitting. The reference values for Ti^3+^ and Ti^4+^ are determined from bulk LaTiO_3_ and bulk SrTiO_3_, respectively.

We explore the electronic structure by EELS fingerprint analysis as shown in Figure 3, and extract the Ti-*L*_2,3_ white lines and O-*K* edge spectra separately from eight rectangular areas (Figure 3a). Using the bulk and GB spectra as references, a multiple linear least-squares (MLLS) fit is shown in Figure 3b, c. We fit Ti-*L*_2,3_ reference spectra for lower-valence-state Ti^(4-δ)+^ and Ti (Ti^4+^), respectively, to distinguish *interfacial* Ti (Figure 3b) from *bulk* Ti (Figure 3c). The energy shift of the Ti-*L*_2,3_ peaks and the reduced peak splitting of the t_2g_ and e_g_ states at the GB (spectrum 5 in Figure 3d) compared to the spectra from regions 1–4 and 6–8 in Figure 3d imply an increased occupancy of the 3d orbitals, corresponding to a reduced Ti valence state at the GB.^32^ Concurrently, the peak B of the O-*K* edge at the GB in Figure 3e decreases in intensity, which is associated with a reduction of the Ti valence, the presence of oxygen vacancies, or the absence of Sr columns.^33−35^ In addition, we calculate the valence state of Ti by applying MLLS fitting, yielding a Ti valence state of around 3.75+ at the GB (Figure 3f). We use the Ti-*L*_2,3_ spectra from LaTiO_3_^36,37^ and bulk SrTiO_3_ as references for Ti^3+^ and Ti^4+^, respectively.

**Figure 4:**
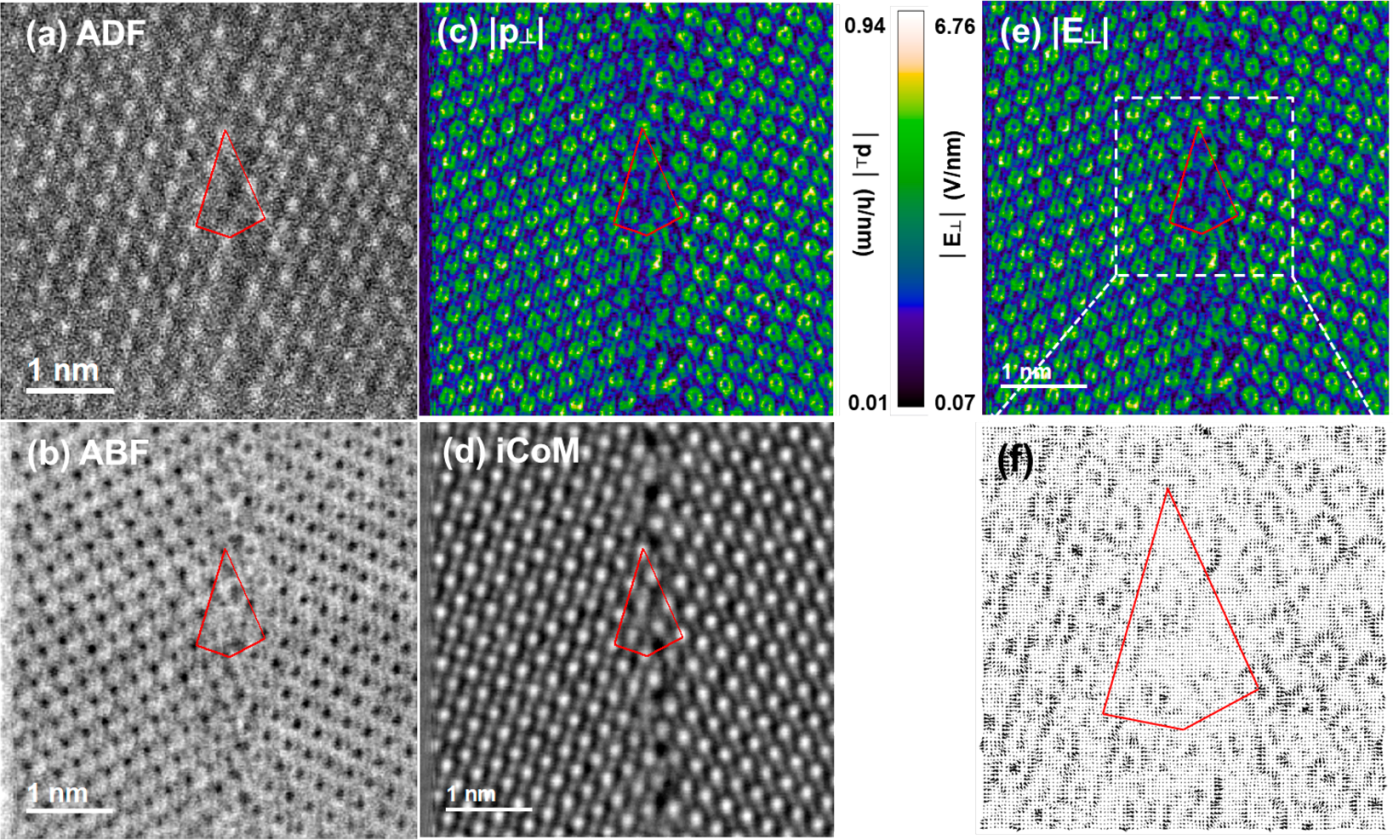
Extracted information from a 4D data set of a Σ5 GB in STO. The electron probe is in the under-focus condition. Reconstructed atomic-column-resolved (a) ADF and (b) ABF by virtual detectors. (c) Magnitude of the average momentum transfer around the GB. (d) Reconstructed iCoM image based on the py4DSTEM library. (e) Electric field derived from the average momentum transfer. (f) Corresponding quiver plot of the electric field from the region marked with white dashed box in panel e, characterizing its strength and rotational symmetry.

Finally, we conduct 4D-STEM experiments to further analyze the GB structure and the electrostatic characteristics. For the 4D-STEM measurements, a pixelated detector records a 2D diffraction pattern at each probe position when the electron probe is scanned across the specimen, generating a 4D data set. Figure 4a, b shows the reconstructed ADF and ABF images, respectively. We calculated the integrated center of mass (iCoM) image (Figure 4d) using the py4DSTEM python library,^38^ which shows a higher contrast of the oxygen columns. It is sensitive to the beam deflection by detecting the center of mass of the electron-intensity distribution. The atomic structure of the GB core in the reconstructed phase image is consistent with that in conventional ABF images (Figure 3), but with a better contrast of the oxygen columns. Partial occupancies of O columns resulting in oxygen vacancies and structural distortions cause a slight contrast decrease. Because of the excess Ti and oxygen vacancies, the GB is positively charged, which is expected to induce negative space-charge zones.^17^

To obtain insight into the electric field in the GB region, we performed calculations based on the simplified quantum mechanical model^10,11^
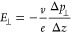
where *E*_⊥_ includes lateral electric-field components *E*_*x*_ and *E*_*y*_, which are proportional to the change of the momentum of the electron beam (Δ*p*_⊥_). *E*_⊥_ relates to the shift of the center of mass (ΔCOM) of each diffraction pattern, Δ*z* is the sample thickness, *e* is the elementary charge, and ν is the electron velocity. Theoretical and simulation studies pointed out that the sample thickness should be below 5 nm for quantitative atomic electric field measurements.^10,39^ However, because of specimen preparation, such thin samples can hardly be regarded as bulk-like, as the surface-near regions (few nanometers) are always nonstoichiometric. Therefore, we selected regions with a thickness of around 10–20 nm, which will give qualitative results of the electric field.^40^ It would be more accurate to perform the measurements of atomic electric fields under the condition that the electron probe is focused on the midplane or below the midplane of the specimen.^40^ We performed the experiments with different focus conditions. First, the constructed images in Figure 4 was performed in the under-focus condition. The given intensity bar in the atomic electric field maps indicate the relative intensity. From the magnitude of the average momentum transfer (Figure 4c), we calculate the magnitude of the electric field (Figure 4e) and the corresponding quiver plot (Figure 4f), where the length and the direction of the black arrows indicate magnitude and direction of the electric field, respectively. From Figure S1, sample thickness for this area is around 16 nm by comparing the experimental and simulated CBED patterns. Generally, the electric field surrounding the cation columns points radially outward and appears approximately rotationally symmetric in the bulk region. However, at the GB, the fields are no longer radially symmetric and the field magnitude changes clearly as compared to the bulk region. As shown in Figure S1, we find a very small tilt for the two adjacent grains by comparing the CBED pattern or the oxygen columns at the bulk region from the iCoM image, which would contribute to a change in the symmetry of atomic electric fields at the grain boundary. As shown in Figure S2, we notice that the strength of momentum transfer at the grain boundary is much stronger than that in the bulk region when the electron probe is focused on the entrance surface of the sample. The obvious difference in momentum transfer under different focus conditions indicates the nonignorable effects of diffraction contrast on the atomic electric field. Therefore, it is hard to disentangle purely geometric and charge effects on the observed variation in electric field at the grain boundary. However, the grain boundary is expected to be positively charged considering the accumulation of positively charged defects, e.g., oxygen vacancies and reduced Ti atoms.

**Figure 5:**
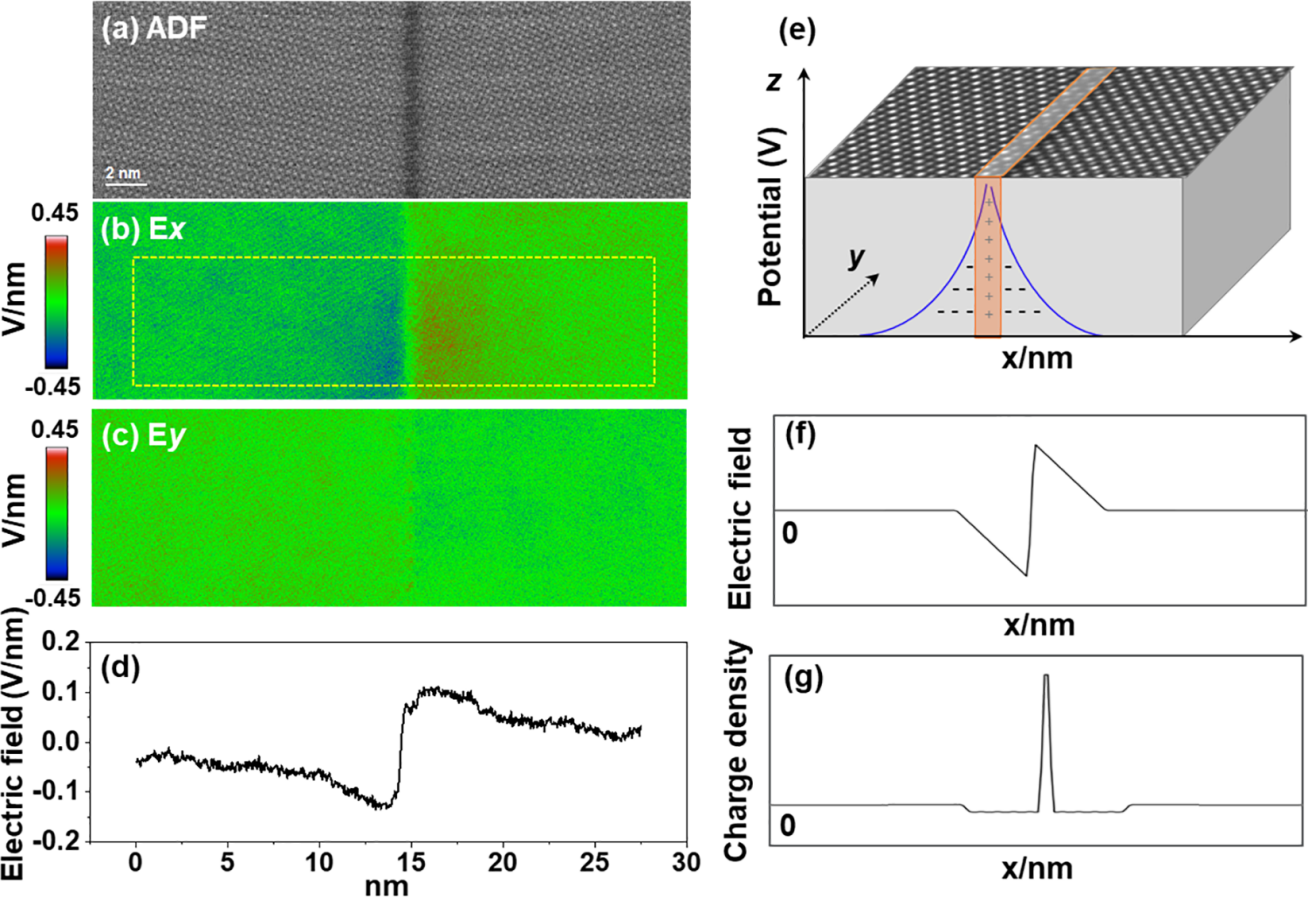
Electrostatic characteristics of the Σ5 GB at a larger scale. (a) ADF image of the GB. Corresponding (b) horizontal direction and (c) vertical direction of the electric field derived from the 4D data set. (d) Line profile of the electric field across the GB. (e) Schematic diagram of the double-Schottky-barrier model with a positive potential at the GB. (f) Corresponding electric field and (g) charge density profiles from the electrostatic potential in the double-Schottky-barrier model.

Given the principle of electrical neutrality, the accumulation of positive charges at the GB should be compensated by a negative charge on both sides of the GB. According to the double-Schottky-barrier model for space-charge formation, the negative charge formed in the bulk region can compensate the positive charge at the GB core. To verify this, we analyzed the electric field on a larger scale, as shown in Figure 5, where an ADF image constructed from the 4D data set (Figure 5a) and the corresponding electric field image (Figure 5b) are displayed. Compared with the atomic electric field, the measurements of the electric field at large scale are less sensitive to the thickness, e.g., the momentum transfer can be averaged over a part of the crystal where the momentum transfer derived from the atomic fields can be compensated because of symmetry.^39^ The *x*-component of the electric field across the grain boundary in Figure 5b shows a distinct difference, whereas the *y*-component of the electric field almost maintains a homogeneous contrast of the whole region as shown in Figure 5c. According to the double-Schottky-barrier model in Figure 5e, the corresponding electric field (Figure 5f) and charge density (Figure 5g) line profiles can be obtained from the Poisson equation. The experimentally measured result in Figure 5d is in good agreement with the double-Schottky-barrier model, suggesting that the positive charge accumulated at the GB core is compensated by a negative charge in the bulk region. Beside the characteristics of the GB itself, imaging the electric field on a larger scale can be affected by other factors. Typically, a variation in the sample thickness can induce a significant change in the measured electric field.^10^ Therefore, we carefully selected an area with a homogeneous thickness for our measurements. As demonstrated in Figure S3, the bulk region adjacent to the GB is almost flat from the line profile of the ADF image across the grain boundary. In addition, the weak contrast at the GB in the ADF image could in principle be due to a thickness difference between the GB and the bulk region. Therefore, we measured an EELS thickness map across the GB (Figure S4), which confirms a uniform thickness across the GB.

## Discussion

From the electric field maps, we can comprehensively identify the electrostatic characteristics of the GB. From the local atomic electric field imaging, we observe a difference of the electric field for the individual atomic columns around the GB core. The contributions to the measured electric field at the grain boundary include a decrease in the Ti valence, accumulation of cations, charged defects, and the change in diffraction contrast. In principle, a decreased Ti valence at the GB can lead to a higher electric field due to an increase in electrons outside of the nucleus. However, by changing the focus condition, we find that the change in diffraction contrast due to the structural disorder and lattice defects affects the measured atomic electric field, which covers the information from charge effects. The observed asymmetric distribution of the measured electric field around atoms is associated with the atomic structure of the GB. On the one hand, the distorted and incomplete oxygen octahedra in the grain-boundary core indicate the structural disorder along the *z*-direction, easily leading to the asymmetric field distribution around cation atoms. Furthermore, because of the close vicinity of O atoms in the GB core, we observe a rigid-body-translation of 0.05 nm along the ⟨310⟩ GB plane, which contributes to the asymmetric field distribution. With respect to the nanometer-scale electric field, we can determine more general information about the electrostatic properties of the GB by averaging over larger regions. We verify that a negative space charge in the bulk region compensates a positive charge accumulated at the GB core, most likely caused by a reduced number of oxygen vacancies in this space-charge area. This is in good agreement with the double-Schottky-barrier model. Thus, a simultaneous characterization of the atomic structure and the atomic-scale as well as micrometer-scale electrostatic characteristics benefits the understanding of the relationship between the atomic structure and the electrostatic properties of the GB.

According to the double-Schottky-barrier model, the space-charge-layer width calculated from impedance-spectroscopy data is much wider than the GB width. For example, the space-charge-layer width of a SrTiO_3_ bicrystal doped with some hundred parts per million Fe is in the range of 30–70 nm with a space charge potential of around 0.6 V,^20,41^ whereas the space-charge-layer width should be even larger for undoped SrTiO_3_.^17^ In this work, we provide evidence of a positive charge accumulation in the GB core and a space-charge layer adjacent to the GB. The width of the space-charge layer appears to be smaller than those mentioned above, measured by impedance spectroscopy. This could be related to the uncertainty of the doping level in our specimen. Another reason for this discrepancy could be the limited sensitivity of electric-field measurements in the TEM. This could lead to the invisibility of the long tail of the space-charge potential far from the GB and thus lead to an apparently narrow zone.

## Conclusions

In summary, we systematically investigated the atomic structure and electrostatic characteristics of a high-angle Σ5 GB in a SrTiO_3_ bicrystal by combining 4D-STEM and atomically resolved STEM-EELS. We demonstrated that mainly Ti occupies most of the atomic sites in the GB core with a minor amount of Sr intermixing. The accumulation of cations and charged defects, e.g., oxygen vacancies, the reduction of Ti, and the structural distortion induced the observed variation in the local electric field. The overall electrostatic characteristic of the GB is in agreement with the double-Schottky-barrier model, revealing that the negative charge arises from a space charge in the bulk to compensate the positive charge in the GB core.

## Methods

A Σ 5 (310) [001] SrTiO_3_ bicrystal was obtained from Shinkosha Co., Ltd. (Tokyo, Japan). The bicrystal possesses tiny amounts of acceptor impurities (around 60 ppm), e.g., Fe, Cr, and Al, as determined by inductively coupled plasma-optical emission spectroscopy (ICP-OES) analysis and shown in Table S1. These acceptor dopants can induce a formation of oxygen vacancies according to the following formula
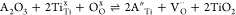
where A stands for trivalent impurities. In addition, because of the intrinsic Schottky disorder reaction
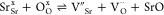
the concentration of frozen-in oxygen vacancies (around 56 ppm) can be estimated from the Schottky equation (assuming it is frozen-in at 1200 °C).^42^ Thus, we calculate a Debye length to be around 3.1 nm. The Mott–Schottky space-charge-layer width is around 69 nm (using the space charge potential of around 0.8 V calculated from Figure 5d). This depletion width is on the same order of magnitude as the space-charge width visible in Figure 5; the deviation can be attributed to the uncertainty in the actual defect concentrations (actual freezing temperature of Schottky reaction, etc.). The TEM specimen was prepared by combining dimple grinding and Ar-ion milling. The Ar-ion-milling process is carried out at liquid-nitrogen temperature with a Gatan precision ion polishing system (Gatan PIPS, model 691).

The STEM investigations were performed on a spherical aberration-corrected microscope (JEOL ARM200F, JEOL Co. Ltd.) equipped with a DCOR probe corrector (CEOS GmbH) and a Gatan GIF Quantum ERS K2 spectrometer at 200 kV. The convergence semiangle of the electron probe was 20.4 mrad for STEM imaging, and 6 mrad for large-scale electric field measurement. The range of collection semiangles for HAADF imaging and ABF imaging were 70–300 mrad and 10–20 mrad, respectively. Spectrum images were acquired at a dispersion of 0.5 eV per channel with an energy resolution of 1 eV and denoised afterward by the principle component analysis (PCA) method.^43^ A Merlin direct electron detector (256 × 256 pixels, Quantum Detectors) was used to record the diffraction patterns at each probe position. In the 4D-STEM experiments, the detector was operated in the 1-bit mode with continuous reading/writing at a pixel dwell time of 4.8 × 10^–5^ s, yielding a frame rate of 20833 fps.

## Supporting Information Available

ICP-OES analysis of impurities and detailed studies of STO bicrystal (PDF)
