# Supplementary material for: Determination of Grain-Boundary Structure and Electrostatic Characteristics in a SrTiO3 Bicrystal by Four-Dimensional Electron Microscopy
Source: Nano Lett. 2021 Oct 21;21(21):9138–45. doi: 10.1021/acs.nanolett.1c02960 (PMC8587898; doi:10.1021/acs.nanolett.1c02960)
Supplement: Supplementary file 1 — nl1c02960_si_001.pdf [file nl1c02960_si_001.pdf]

Supporting information for

Determination of grain-boundary structure and electrostatic characteristics in a $SrTiO_3$ bi-crystal by four-dimensional electron microscopy

Chao Yang[1] *, Yi Wang[1,2] *, Wilfried Sigle[1], Peter A. van Aken[1]

[1] Max Planck Institute for Solid State Research, Stuttgart, 70569, Germany

[2] Center for Microscopy and Analysis, Nanjing University of Aeronautics and Astronautics, Nanjing, 210016, P.R. China

* Corresponding authors: c.yang@fkf.mpg.de, wang.yi@nuaa.edu.cn

Table. S1

Host elements	*wt* %
Sr	47.6 ± 0.5
Ti	26.1 ± 0.3
O	25.9 ± 0.3

Impurities	*wt* %	*at%*
Si	0.01	0.066
Ca	0.01	0.046
Ba	0.007	0.009
Cr	0.001	0.004
Mg	0.0001	< 0.001
Fe	0.0001	< 0.001
Al	0.0002	0.001

Table. S1 Impurities concentration in the $SrTiO_3$ bi-crystal ceramic

Fig. S1

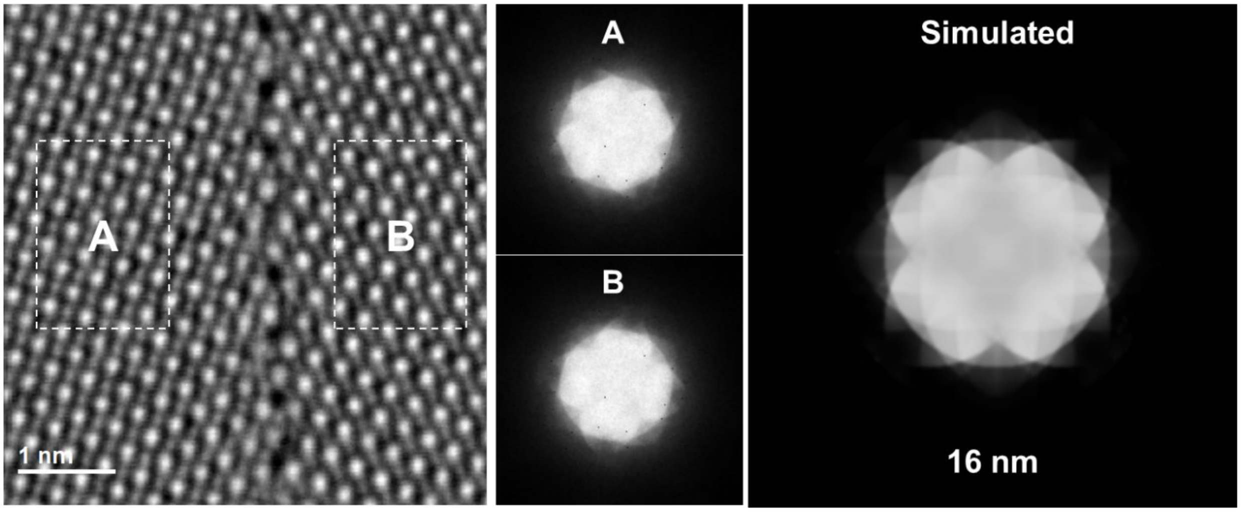

Figure S1. The experimental PACBED patterns extracted from left and right grains at the area marked with white boxes in iCoM image. The simulated CBED pattern calculated from JEMS software.

Fig. S2

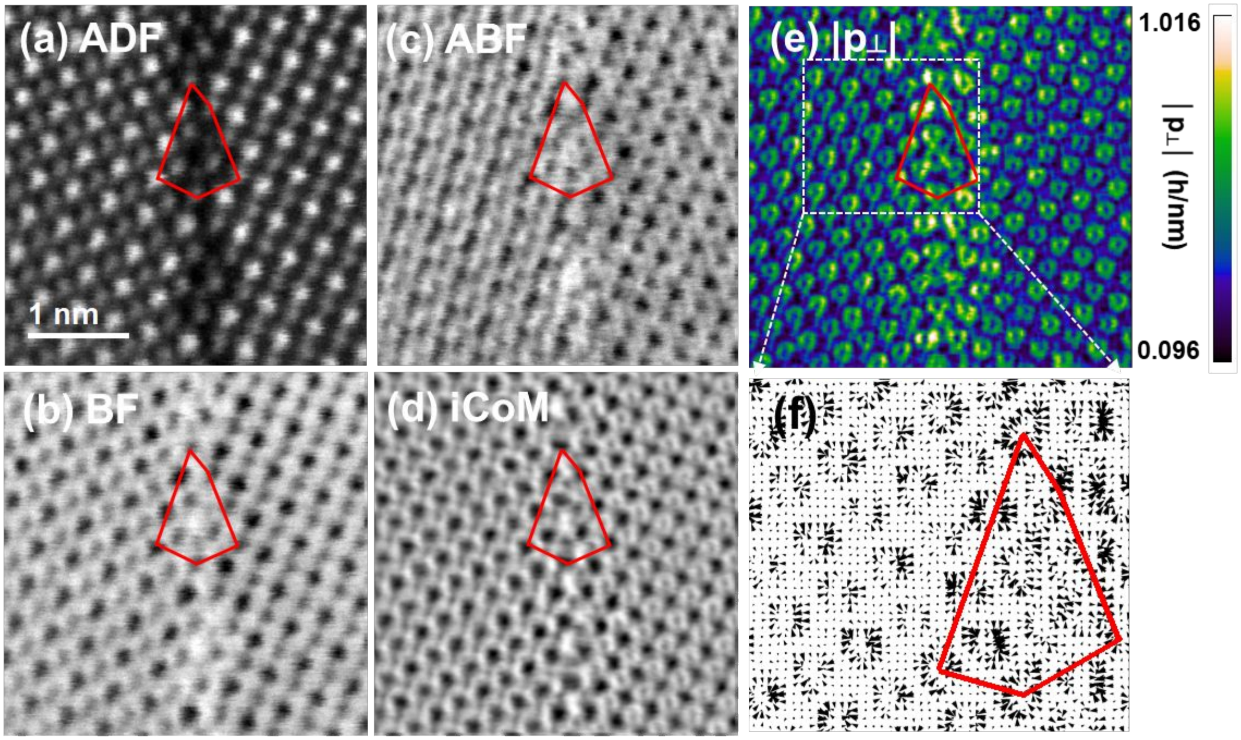

Fig. S2 The extracted information from a 4D dataset of a Σ5 GB in STO. The electron probe is focused on the entrance surface of sample. Reconstructed atomic-column-resolved (a) ADF, (b) BF, and (c) ABF by virtual detectors. (d) The reconstructed iCoM image based on the py4DSTEM library. (e) The magnitude of the average momentum transfer around the GB. (f) The corresponding quiver plot of the average momentum transfer from the region marked with white dashed box in (e), characterizing its strength and rotational symmetry.

Fig. S3

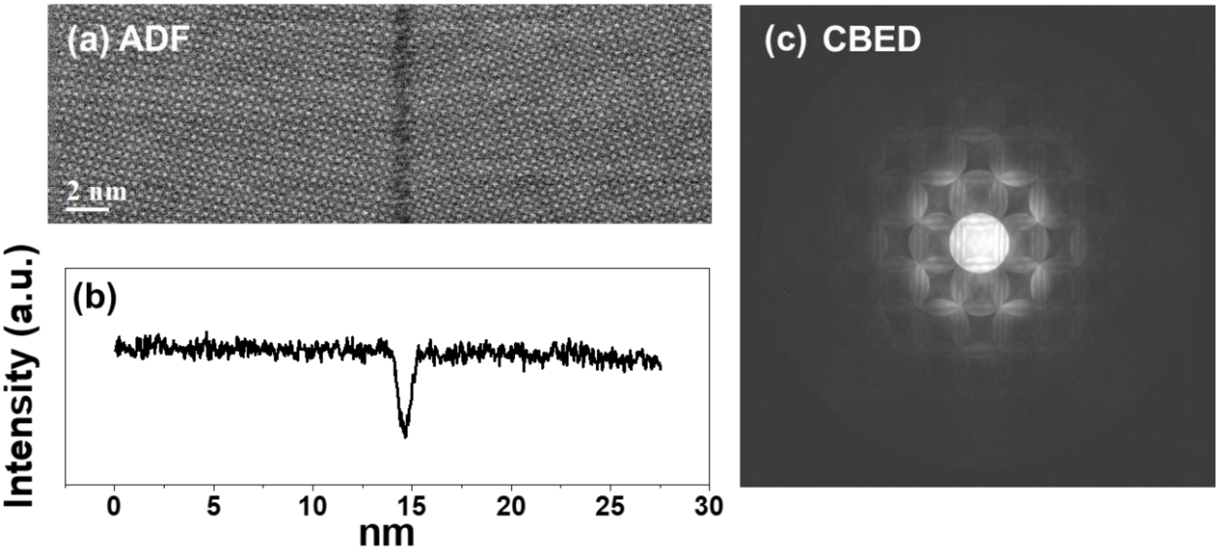

Figure S3. (a) ADF image contrast from 4D-STEM dataset and the corresponding vertical averaged line profile (b). (c) CBED pattern with the convergent angle around 6 mrad.

Fig. S4

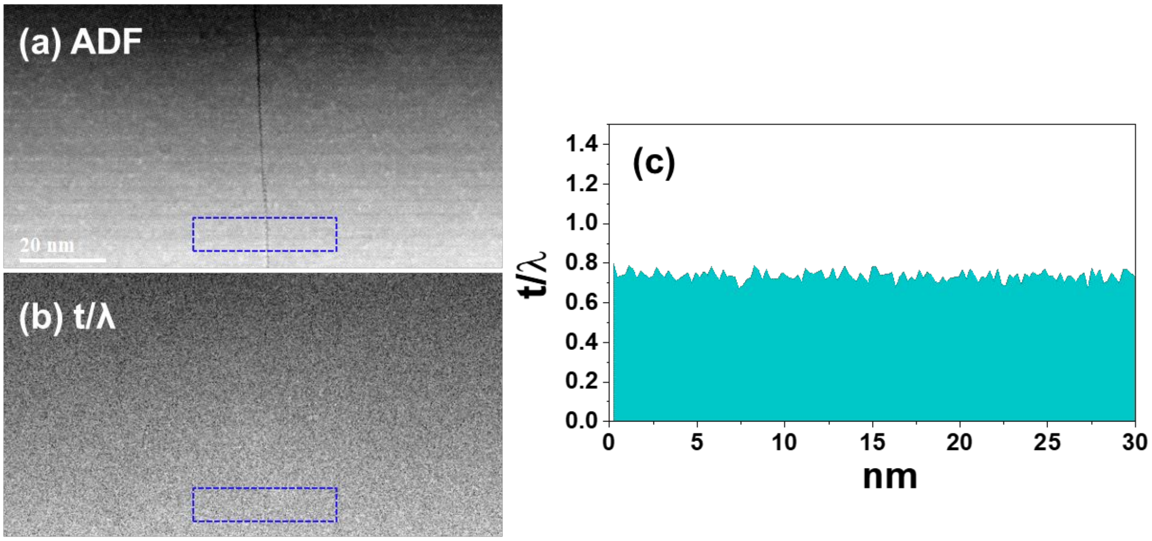

Figure S4. An EELS measurement across the GB. (a) The ADF image for the acquired EELS data. (b) Thickness map. (c) Line profile vertically averaged of the region marked with blue dashed box in (b).
